# Supplementary material for: Developing a taxonomy of care coordination for people living with rare conditions: a qualitative study
Source: Orphanet J Rare Dis. 2022 Apr 20;17:171. doi: 10.1186/s13023-022-02321-w (PMC9020422; doi:10.1186/s13023-022-02321-w)
Supplement: Supplementary file 2 — Additional file 2: Topic guide for workshops. [file 13023_2022_2321_MOESM2_ESM.docx]

## Appendix

Appendix 2. Topic guide for workshop

| Time | Tasks/sessions |
| --- | --- |
| 10 minutes | Introduction to workshop and ground rules & brief intro to participants & brief recap of video/introduce task |
| 40 minutes | Group discussion on taxonomy (domains and characteristics) – go through each domain answering the following questions:   - What’s good about this domain and the characteristics within it? (10 mins) - What needs improving? (10 mins) - Appropriateness of characteristics within this domain in relation to use during current COVID situation? (10 mins) - Recommendations to improve domain/characteristics? (10 mins)   If time left – could also ask similar questions about the models |
| 10 minutes | Development of recommendations to improve taxonomy and models (summary from discussion and any other thoughts?) |
| 5 minutes | Introduce optional activity for after workshop (if they would like to they can provide feedback on models using the following questions:   - What’s good about the model? - What needs improving? - Appropriateness of model in relation to use during current COVID situation? - Recommendations to improve model?) |
| 5 minutes | Questions and summary/debrief |
