# Supplementary material for: Developing a taxonomy of care coordination for people living with rare conditions: a qualitative study
Source: Orphanet J Rare Dis. 2022 Apr 20;17:171. doi: 10.1186/s13023-022-02321-w (PMC9020422; doi:10.1186/s13023-022-02321-w)
Supplement: Supplementary file 3 — Additional file 3: Visual representation of workshop findings. [file 13023_2022_2321_MOESM3_ESM.pdf]

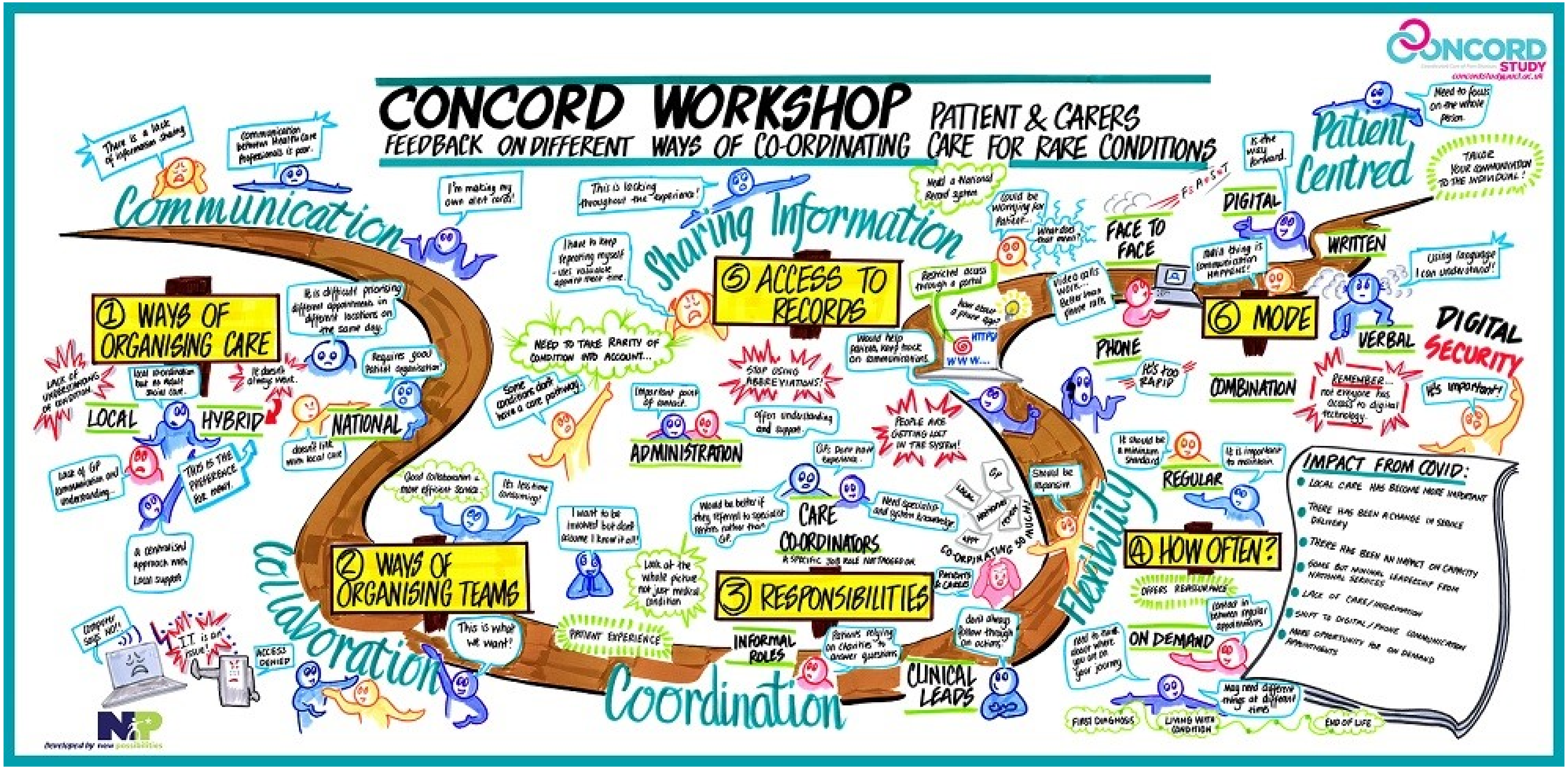
CONCORD WORKSHOP
PATIENT & CARERS
FEEDBACK ON DIFFERENT WAYS OF CO-ORDINATING CARE FOR RARE CONDITIONS
CONCORD STUDY
Communication
There is a lack of information sharing
Communication between Health Care Professionals is poor.
I'm making my own alert cards!
This is lacking throughout the experience!
Sharing Information
I have to keep repeating myself - uses valuable appointment time.
1 WAYS OF ORGANISING CARE
It is difficult prioritising different appointments in different locations on the same day.
Requires good patient organisation!
LACK OF UNDERSTANDING OF CONDITION
It doesn't always work.
LOCAL
HYBRID
NATIONAL
doesn't link with local care
Lack of GP communication and understanding...
THIS IS THE PREFERENCE FOR MANY.
A centralised approach with Local support
NEED TO TAKE RARITY OF CONDITION INTO ACCOUNT...
Some conditions don't have a care pathway.
5 ACCESS TO RECORDS
Important point of contact.
ADMINISTRATION
Offer understanding and support.
STOP USING ABBREVIATIONS!
Need a National Record system
Could be worrying for Patient...
What does that mean?
Restricted access through a portal
How about a phone app?
HTTPS
WWW...
Would help patients keep track on communications.
PEOPLE ARE GETTING LOST IN THE SYSTEM!
FACE TO FACE
Video calls work... Better than phone calls.
PHONE
It's too RAPID
DIGITAL
Is the way forward.
WRITTEN
6 MODE
VERBAL
COMBINATION
Using language I can understand!
Patient Centred
Need to focus on the whole person.
TAILOR YOUR COMMUNICATION TO THE INDIVIDUAL!
DIGITAL SECURITY
REMEMBER... not everyone has access to digital technology.
It's important!
Good collaboration = more efficient service.
It's less time consuming!
2 WAYS OF ORGANISING TEAMS
I want to be involved but don't assume I know it all!
Look at the whole picture not just medical condition
This is what we want!
PATIENT EXPERIENCE
Collaboration
Computer says NO!
I.T. is an issue!
ACCESS DENIED
Would be better if they referred to specialist centres rather than GP.
CARE CO-ORDINATORS
Need specialist and system knowledge.
CO-ORDINATING SO MUCH!
3 RESPONSIBILITIES
Patients & Carers
INFORMAL ROLES
Patients relying on charities to answer questions
Don't always follow through on actions!
CLINICAL LEADS
Coordination
Should be responsive.
Flexibility
It should be a minimum standard.
REGULAR
It is important to maintain.
4 HOW OFTEN?
OFFERS REASSURANCE
Contact in between regular appointments
ON DEMAND
May need different things at different times
FIRST DIAGNOSIS
LIVING WITH CONDITION
END OF LIFE
IMPACT FROM COVID:
LOCAL CARE HAS BECOME MORE IMPORTANT
THERE HAS BEEN A CHANGE IN SERVICE DELIVERY
THERE HAS BEEN AN IMPACT ON CAPACITY
SOME BUT MINIMAL LEADERSHIP FROM NATIONAL SERVICES
LACK OF CARE / INFORMATION
SHIFT TO DIGITAL / PHONE COMMUNICATION
MORE OPPORTUNITY FOR ON DEMAND APPOINTMENTS
Developed by new possibilities

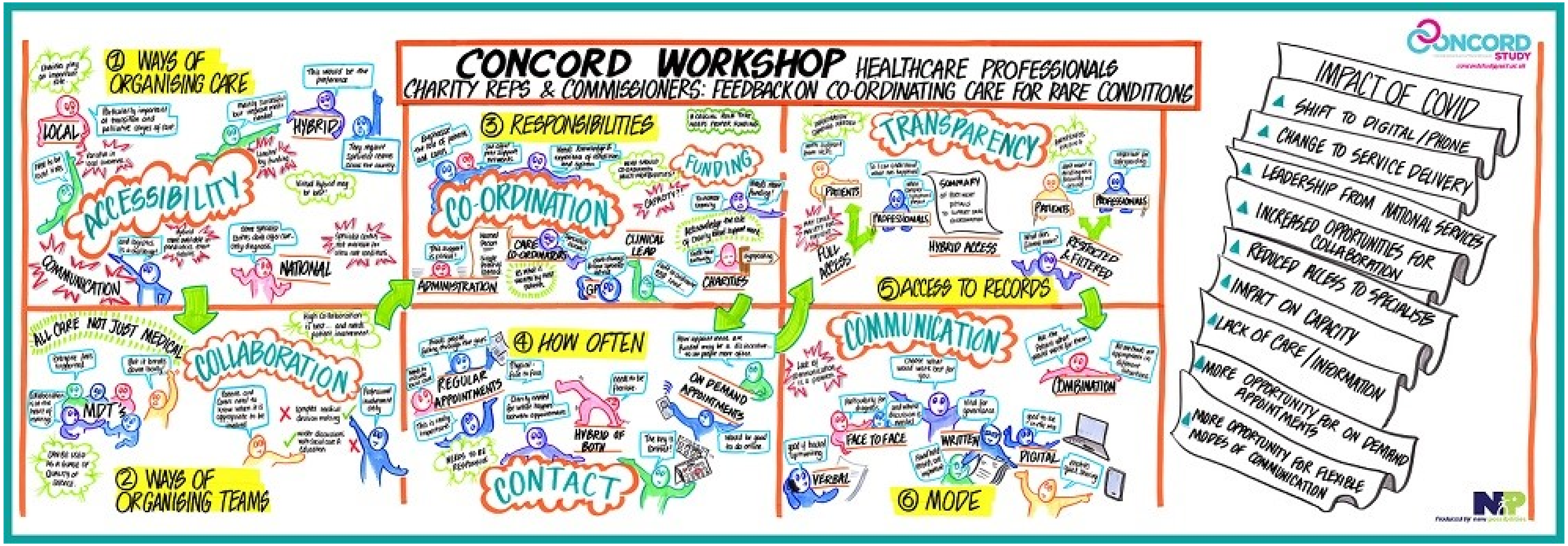

CONCORD WORKSHOP HEALTHCARE PROFESSIONALS
CHARITY REPS & COMMISSIONERS: FEEDBACK ON CO-ORDINATING CARE FOR RARE CONDITIONS
CONCORD STUDY
① WAYS OF ORGANISING CARE
LOCAL
HYBRID
ACCESSIBILITY
COMMUNICATION
NATIONAL
② WAYS OF ORGANISING TEAMS
ALL CARE NOT JUST MEDICAL
COLLABORATION
MDT's
③ RESPONSIBILITIES
FUNDING
CO-ORDINATION
CARE CO-ORDINATORS
CLINICAL LEAD
ADMINISTRATION
GP
CHARITIES
④ HOW OFTEN
REGULAR APPOINTMENTS
HYBRID OF BOTH
ON DEMAND APPOINTMENTS
CONTACT
TRANSPARENCY
PATIENTS
PROFESSIONALS
SUMMARY
FULL ACCESS
HYBRID ACCESS
RESTRICTED & FILTERED
⑤ ACCESS TO RECORDS
COMMUNICATION
COMBINATION
FACE TO FACE
WRITTEN
DIGITAL
VERBAL
⑥ MODE
IMPACT OF COVID
SHIFT TO DIGITAL / PHONE
CHANGE TO SERVICE DELIVERY
LEADERSHIP FROM NATIONAL SERVICES
INCREASED OPPORTUNITIES FOR COLLABORATION
REDUCED ACCESS TO SPECIALISTS
IMPACT ON CAPACITY
LACK OF CARE / INFORMATION
MORE OPPORTUNITY FOR ON DEMAND APPOINTMENTS
MORE OPPORTUNITY FOR FLEXIBLE MODES OF COMMUNICATION
